# Population characteristics and self-assessment of speaking and singing voice in Polish contemporary commercial music singers—an exploratory, cross-sectional study

**DOI:** 10.3389/fpubh.2024.1256152

**Published:** 2024-05-15

**Authors:** Joanna Morawska, Wioletta Pietruszewska, Piotr Politański, Ewa Niebudek-Bogusz

**Affiliations:** ^1^Department of Otolaryngology, Head and Neck Oncology, Medical University of Lodz, Łódź, Poland; ^2^Department of Electromagnetic Hazards, Nofer Institute of Occupational Medicine, Łódź, Poland

**Keywords:** contemporary commercial music singers, demographic characteristics, professional voice, voice disorders, self-assessment, singing voice handicap index, vocal tract discomfort scale

## Abstract

**Background:**

The domination of the Contemporary Commercial Music (CCM) industry in music markets has led to a significant increase in the number of CCM performers. Performing in a wide variety of singing styles involves exposing CCM singers to specific risk factors potentially leading to voice problems. This, in turn, necessitates the consideration of this particular group of voice users in the Occupational Health framework. The aim of the present research was threefold. First, it sought to profile the group of Polish CCM singers. Second, it was designed to explore the prevalence of self-reported voice problems and voice quality in this population, in both speech and singing. Third, it aimed to explore the relationships between voice problems and lifetime singing involvement, occupational voice use, smoking, alcohol consumption, vocal training, and microphone use, as potential voice risk factors.

**Materials and methods:**

The study was conducted in Poland from January 2020 to April 2023. An online survey included socio-demographic information, singing involvement characteristics, and singers’ voice self-assessment. The prevalence of voice problems was assessed by the Polish versions of the Vocal Tract Discomfort Scale (VTDS) and the Singing Voice Handicap Index (SVHI). Also, a self-reported dysphonia symptoms protocol was applied. The perceived overall voice quality was assessed by a Visual Analogue Scale (VAS) of 100 mm.

**Results:**

412 singers, 310 women and 102 men, completed the survey. Nearly half of the studied population declared lifetime singing experience over 10 years with an average daily singing time of 1 or 2 h. 283 participants received vocal training. For 11.4% of respondents, singing was the primary income source, and 42% defined their career goals as voice-related. The median scores of the VTDS were 11.00 (0–44) and 12.00 (0–40) for the Frequency and Severity subscales, respectively. The median SVHI score of 33 (0–139) was significantly higher than the normative values determined in a systematic review and meta-analysis (2018). Strong positive correlations were observed between SVHI and both VTD subscales: Frequency (*r* = 0.632, *p* < 0.001) and Severity (*r* = 0.611, *p* < 0.001). The relationships between most of the other variables studied were weak or negligible.

**Conclusion:**

The examined CCM singers exhibited substantial diversity with regard to musical genre preferences, aspirations pertaining to singing endeavors, career affiliations, and source of income. Singing voice assessment revealed a greater degree of voice problems in the examined cohort than so far reported in the literature, based on the SVH and VTDS.

## 1 Introduction

Contemporary Commercial Music (CCM) is a term coined by Jeannette LoVetri (1) and refers to all non-classical, non-lyrical, and popular styles of singing. These include a wide variety of sub-genres and styles such as pop, rock, rhythm and blues, jazz, hip-hop, country, and heavy metal (2). CCM has become one of the most important cultural phenomena in the 20th and 21st centuries. With the invention of broadcasting media and new recording systems, the mass consumption of recorded music has increased making it one of the most fruitful entertainment businesses today (3). The Nielsen Music Year-End Report (2018) revealed that classical music only accounted for 1% of all music consumed in the US in 2017 (4). Other industry data on ticket sales for live performances confirm that consumer spending on musical theater and other CCM shows is considerably higher than on classical performances (5).

The Polish music market does not differ greatly from foreign markets. In 2019 a comprehensive study of the music market was carried out at the request of the Ministry of Culture and National Heritage. The report by Sokołowski et al. concludes that similar trends to those present globally can also be seen in Poland: increasing value of the music market and growing share of independent labels in the phonographic industry. The value of music sales on the Polish market in 2018 amounted to PLN 330 million (Polish currency). The authors point to the growing popularity of pop, which is also one of the three most popular music genres—Poland’s best-selling record charts were dominated by pop music and hip hop. The research also shows that 44% of respondents declared participation in concerts (6).

The domination of the CCM industry in music markets has led to a significant increase in the number of performers of CCM across its diversity of styles. Performing in a wide variety of singing styles involves exposing CCM singers to specific risk factors that might lead to voice problems in this population. This, in turn, necessitates the consideration of this particular group of voice users in the Occupational Health framework.

Historically, classical singers have received more attention and have been the subject of investigation regarding vocal function and vocal health (7–9). Since 2010 a new body of literature focusing on musical theater and contemporary commercial music (CCM) genres has emerged (10, 11), and more studies have focused on vocal function and health in non-classical styles of singing. However, the needs of CCM singers have not yet been addressed as comprehensively as those of classical singers have been (7).

It is worth underlining at this point that classical and non-classical singing styles differ in several aspects. The main difference is that these two singing styles use different vocal tract, articulators, and, breathing pattern configurations to achieve stylistic requirements (1). Classical singing requires vocal quality with harmonic richness, appropriate articulatory control, and vocal projection. In this way, even without the use of electronic amplification, the voice can be heard over the loud orchestra (12). Active control of the abdomen, a stable and relatively low positioning of the larynx, a raised soft palate, a proper resonance strategy, a consistent vibrato, and tall and rounded vowels are important aspects of the classical singing style (13). In Contemporary Commercial Music, in turn, the most commonly observed characteristics are chest voice dominance, little to no vibrato, vocal registers that are distinct rather than blended, intentional use of noise, irregular vibrations, breathiness, and nasality in the vocal tone (13–15).

The vocal demands on the singer’s voice are multifaceted. Oftentimes, professional singers use their voices at their maximum effort level and are exposed to unique vocal demands, including high environmental and performance demands (16). These demands are far more than what an average individual places on the voice through moderate daily speech (17) and the activity of singing requires more endurance, flexibility, and vocal tract control (18). This has given singers the special status of the most demanding vocal group – elite vocal performers among all voice users (10, 19). Continuous vocal production is an activity involving a synchronized interaction of multiple physical processes such as respiration, phonation, and resonance (20, 21). This, in turn, exposes singers’ voices to elevated risk factors, both ergonomic (environmental) and extra-occupational (individual) (21). Some of these factors concern all singers, while others are more common in CCM singers. Being exposed to a wide range of risk factors places singers at an increased risk of laryngeal pathologies and symptoms associated with vocal effort and vocal fatigue (22). Deterioration in voice production may significantly impact the quality of life when a singer’s voice is affected by organic or functional disorders (23).

Numerous studies underline that maintaining vocal hygiene in vocal professionals contributes to vocal health and that lack of knowledge on this subject is one of the frequent risk factors for developing a voice disorder (24). Vocal hygiene includes behaviors such as drinking plenty of water, limiting coffee-tea consumption, monitoring sleep quality, maintaining ambient air humidity, avoiding prolonged periods of voice use, reducing smoking and alcohol consumption, and avoiding medications and other treatments that impair the voice (25). Unlike CCM singers, classical singers typically receive recommendations on proper vocal hygiene from teachers, speech-language pathologists, and laryngologists hoping that they can avoid vocal problems through preventative lifestyle modification (26). However, it should not be assumed by a clinician that a singer of one genre has the health information relevant to their voice problem without further inquiry and probes (27).

According to literature data, the control of several comorbidities such as reflux, allergy, or nasal disorders is crucial for maintaining good quality of the voice. It has been reported that laryngopharyngeal reflux disease (LPR) is associated with an increased number of voice symptoms (28). Singers are particularly exposed to the risk of presenting LPR because of necessary air support involving intense use of abdominal muscles, higher intra-abdominal pressure, increased stress due to career management and uncomfortable schedules, late meals just before sleep, bad nutrition habits like increased intake of citrus products, fat and spicy foods (28). Allergy should also be considered as an underlying factor for vocal symptoms, especially for persons who work in vocally demanding occupations (29). Nasal and paranasal sinus diseases may alter the quality of voice and voice performance (30).

Among other factors that contribute to a higher risk of developing voice disorder in CCM singers are extra-occupational vocal activity and engaging in misuse or overuse of voice in “the day job” (31) as well as off-stage behavior sometimes characterized by lack of vocal and physical rest (32). Therefore, in singers’ voice assessments examining singing voice quality as well as speaking voice quality is necessary to obtain a comprehensive picture of the pathomechanism of the disorder (33). Bartlett and Wilson suggest that at least some of the singers’ reported voice problems may be caused by improper or excessive use of the speaking voice rather than an improper use of their singing voice (31).

Vocal training programs are considered helpful in improving vocal capabilities to meet the professional demands of singers as well as to limit potential damage to the voice. Compared to classically trained singers, vocalists performing CCM are generally less likely to have formal voice training or an understanding of voice production mechanics (34).

Through the development of distinct stage personas, singers establish a market for their music. The success and longevity of their careers are often determined by their unique vocal individuality and innovative stylistic approach (7). Voice usage differs considerably among various styles of singing. Certain styles are commonly regarded as potentially harmful to vocal health and phonatory mechanisms, particularly those that use strong glottal adduction and high subglottal pressures (35, 36). Moreover, various singing styles make use of a wide variety of special vocal effects such as Distortion, Rattle, Growl, Grunt, Creaking, Air added to the voice, Screams, Vocal Breaks, and Ornamentation techniques (36).

Psychological problems associated with the profession include worry about possible negative evaluation and fear of vocal indisposition. In a psychosocial aspect, singers’ problems also concern difficulties in maintaining a family life and relations due to irregular working hours, frequent trips, and concert tours.

Other aspects that should be taken into consideration as potentially putting additional strain on the voice of singers are environmental factors such as poor room acoustics, singing in environments with high levels of background noise, and poor air quality. Additionally, the demands placed upon the CCM singers by the entertainment industry can also contribute to the development of voice disorders in this population (37).

The interest in singing has been growing since the first indexed paper in PubMed was published in 1949. Currently, the demands of singing voice use play an important role in clinical research (18). Even though research examining contemporary commercial music styles of singing has increased significantly over the last 20 years (38), surveys on the prevalence of voice disorders in singers are scarce in the literature (39). In a systematic review and meta–analysis Pestana et al. in 2017 found the overall prevalence of self-reported dysphonia was 40.53% in classical and 46.96% in nonclassical singers (10). The data in this research included original papers written in English, Portuguese, or Spanish, and published in peer-reviewed journals.

Compared to non-singers, singers may be more sensitive to vocal symptoms and experience a different quality of life impact (40). Furthermore, they perceive a marked distinction between talking and singing and may have general complaints about the voice and specific complaints that relate only to the singing voice (41).

The growing interest in the singing voice has induced a lot of research concerning voice assessment focusing on qualitative and quantitative aspects of vocal performance. The available research on the prevalence of voice disorders in singers shows that there is no methodological uniformity in the research on the vocal performance of singers. There exists a variety of research methodologies that can be applied. A great number of publications present data derived from self-reported methodology (10, 26, 42–44). In other cases, self-assessment of voice is combined with videostrobolaryngoscopy (11, 17). In a recent study, Kazi et al. assessed vocal health in singing students with the use of cepstral peak prominence (CPP) to provide preliminary information on this measure (45). Timmermans et al. assessed the voice quality of the study participants employing a multi-dimensional test battery which was in line with the recommended protocol for diagnosing voice disorders. It states that a comprehensive voice evaluation should include a visual examination of the larynx, perceptual assessment of vocal quality, aerodynamic measures, acoustic analysis, and vocal self-assessment procedures (46, 47). It should be noted, though that each of the methods mentioned has a specific relevance and can provide particular information.

Deterioration in voice production can be debilitating, physically and psychologically (21), and can significantly impact the quality of life when a singer’s voice is affected by organic or functional disorders (23). Despite a great number of risk factors related to singing and CCM singers’ tendency toward voice problems, these voice professionals rarely seek medical help for a voice disorder until it progresses into a severe pathology (48). This seems to be true also for the population of Polish CCM singers. In a study in 2019, Sielska-Badurek et al. compared the prevalence of vocal fold pathologies among first-year singing students from the classical, musical theater, and contemporary commercial music (CCM) genres. They reported that CCM and musical theater first-year students had a significantly higher prevalence of vocal fold pathology compared to first-year students of classical singing who were found to have no vocal fold pathology. No other research on voice disorders in the population of Polish CCM singers has been found by the authors of this study. Given that CCM is the predominant singing style globally, prevention and effective management of voice disorders is an important concern to all professionals who are involved in the assessment and management of vocal health in this population of voice users.

The purpose of this study was threefold: to characterize the population of CCM singers in Poland, determine the prevalence of self-reported voice problems in this group, and explore the relationships between the studied variables. The specific research questions were: (1) What are the demographic, occupational, and singing involvement characteristics of CCM singers in Poland? (2) What is the prevalence of self-reported voice problems in speech and singing in this population? (3) What is the relation of the variables of interest: lifetime singing involvement, vocal training received, smoking, alcohol consumption, occupational voice profile, and microphone use to the degree of self-perceived voice problems?

In this study, the assumed definition of a “voice problem” was based on any self-perceived difficulty in voice production, including voice quality changes, the presence of dysphonia, phonatory effort, or any other vocal symptoms.

## 2 Materials and methods

### 2.1 Study design

The study was conducted from January 2020 to March 2023 in Poland, targeting the population of Polish CCM amateur and professional singers. An online survey was designed to obtain the data for the study. The survey was designed by the authors of the study, based on a literature review regarding the given topic and the authors’ own clinical experience. Since the nature of the present study considers singers’ self-reports, we selected self-assessment instruments that will be described later in detail.

### 2.2 Participants

The study was conducted on a representative sample whose size was determined^1^ for 95% power of the study and type 1 error probability α of 0.05. The estimated sample size was 385 subjects. The required criteria were satisfied as 412 singers filled out the survey. The final sample comprised 412 participants recruited through convenience sampling. The data presented in this study are part of a longitudinal study on vocal health in CCM singers.

#### 2.2.1 Inclusion criteria

To be included in the study the participants had to be over 18 years old and identify themselves as Contemporary Commercial Music singers. No specific musical or singing style was required. Both professional and amateur singers were invited to take part in the survey. The Informed Consent Form was presented on the screen, and continuing to the next screen was only possible if the volunteer marked the option consenting to participate in the study.

#### 2.2.2 Exclusion criteria

Classical music singers were not included in the study to focus specifically on understudied CCM singers.

### 2.3 Ethical considerations

Approval for this study was granted by the Ethical Committee of the Medical University of Lodz (Decision no. RNN/11/20 KE).

Participants were informed about the goal of the survey (medical research). To limit information bias, all participants were offered the same explanation regarding the nature and the aim of the study in the preface of the questionnaire. The data for this research did not have to be associated with a specific respondent, therefore the survey was anonymous and no personally identifiable data was collected. Respondents received information about the possibility of conducting a comprehensive voice examination at a later stage of the research. Those interested were given the opportunity to leave contact details (telephone number, e-mail) so that researchers could contact them and arrange an appointment, or provide information about which facilities in the area of residence can be contacted for voice examination. The information about the voice examination included a detailed description of the procedures that would be performed: routine ENT examination, laryngovideostroboscopy of the larynx, perceptual voice assessment, High-Speed Videolaryngoscopy, and acoustic analysis of the voice. It was clearly stated that it was addressed to CCM singers. This was an additional option in the survey and lack of interest in participating in the voice examination did not exclude participants. Taking part in the study did not involve any costs incurred by the participants at any stage.

### 2.4 Data acquisition

The online survey on Google was disseminated on singers’ commonly used social media platforms: Facebook pages (for amateur and professional singers), websites dedicated to singers, and through word of mouth from the investigators to faculty and colleagues. In an attempt to achieve normal distribution and a representative sample, we asked the participants to share the online survey with their peers.

### 2.5 Online survey

**Figure 1:**
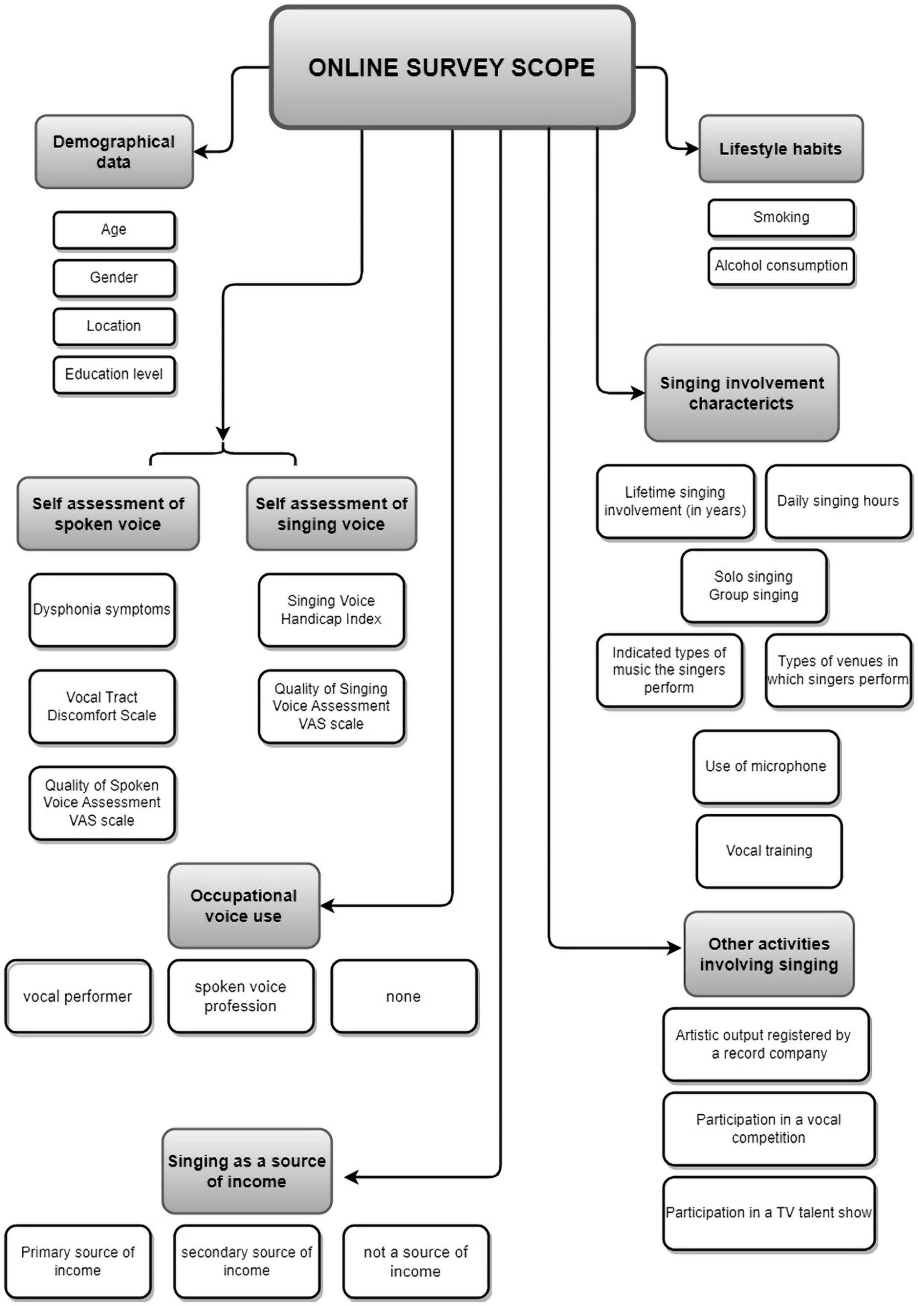
The scope of an online questionnaire for contemporary commercial music singers.

The online survey was divided into several sections, each dealing with a separate aspect of singing: demographical data and lifestyle habits, singing involvement characteristics, voice-related occupational status, and self-assessment of speaking and singing voice. Single-ease questions were used to elicit concise and uncomplicated responses. A flowchart on the scope of the survey is presented in Figure 1.

#### 2.5.1 Demographical data and lifestyle habits

Demographical data query included age, gender, highest education level completed at the time of the survey, and location: urban, suburban, rural. The respondents were asked about current and former tobacco use and alcohol consumption.

#### 2.5.2 Singing involvement characteristics

Singing Involvement section sought to elicit information on lifetime singing involvement, daily amount of time devoted to singing, types of music performed, venues in which the singers typically perform, the use of a microphone during performances, and whether the singers mainly engage in solo or group singing. The participants were also asked whether they had received any vocal training throughout their singing careers. The surveyed singers provided information on whether their artistic output was registered by a record company and is available for sale. Additional information collected in the survey concerned the singers’ participation in singing contests and TV talent shows.

#### 2.5.3 Voice-related occupational status of the participants

This section focused on gathering information on whether the participants’ day jobs involved voice use and if so, to what extent. Additionally, the singers were queried whether singing is their primary, secondary, or no source of income.

#### 2.5.4 Self-assessment of the speaking voice

Self-assessment of the speaking voice was conducted using the Polish version of the Vocal Tract Discomfort Scale (VTDS) (49). The VTDS is a self-assessed questionnaire for the subjective evaluation of voice disorders, proposed by Mathieson in 1993 (50). It is a seven-point self-rating scale that enables patients to indicate the frequency and severity of their vocal tract symptoms on a numerical scale from 0 to 6 (51). The VTDS was chosen because, unlike other voice assessment scales, it can demonstrate many complex aspects of throat symptoms leading to voice disorders (52) and was used to quantify the frequency and severity of an individual’s throat discomfort.

The survey also included information on the frequency of occurrence of typical voice symptoms indicative of dysphonia: hoarseness, dry cough, wet cough, aphonia episodes, shortness of breath, throat clearing, voice breaks, vocal fatigue after voice use, and effortful speaking. The nine analyzed symptoms were selected by the authors based on their own clinical experience and literature data (24, 53). Responses to vocal symptom queries were recorded using a 5-point Likert scale: “never, almost never, sometimes, almost always, always.” A total score of the symptoms was calculated; the scores could range from 0 to 36 points.

Additionally, the patients rated the overall quality of their speaking voice at the moment of the survey on a 10-point analogue VAS scale, where 0 meant very low quality and 10 very high quality, therefore the higher score indicates an individual’s satisfaction with speaking voice quality.

#### 2.5.5 Self-assessment of the singing voice

For the singing voice assessment, the Polish version of the Singing Voice Handicap Index (SVHI) was used. The SVHI questionnaire consists of 36 items that conceptually evaluate the physical, emotional, economic, and social impact of singing voice problems individually graded on a five-point Likert scale (0 = never, 1 = almost never, 2 = sometimes, 3 = almost always, 4 = always) to range from 0 to 144 (54).

The participants were also asked to rate the overall quality of their singing voice at the moment of the survey on a 10-point VAS scale, where 0 meant very low quality and 10 very high quality, therefore the higher score indicates an individual’s satisfaction with singing voice quality.

### 2.6 Statistical analysis

Statistical analysis was performed by means of IBM SPSS Statistics version 20. The results were considered statistically significant if the *p*-value was less than 0.05 (*p* < 0.05). Means, standard deviations, but also medians, and ranges (due to lack of normal variable distribution) were calculated for continuous variables and frequencies and percentages for categorical variables. The results of the VTDS, Dysphonia Symptoms, and SVHI total scores for men and women were compared using the Mann–Whitney U test.

To deal with the parametric and nonparametric variables of the study, Spearman rank correlation (rho) was performed to investigate the correlation between the studied variables: lifetime singing involvement, vocal training received, smoking, alcohol consumption, occupational voice profile, microphone use and the scores of self-assessment scales used in the study: VTDS, Dysphonia symptoms, VAS, speaking voice, SVHI, VAS, singing voice. Similarly, Spearman rank correlations were determined between the scores of all the self-assessment tools used in the study.

## 3 Results

### 3.1 Demographic characteristics and lifestyle habits

The questionnaire garnered a total of 412 responses from singers who met the inclusion criteria. The respondents’ mean age was 29.13 ± 8.67 (mean ± SD), there were 310 women (75.2%), aged 28.28 ± 8.18 (mean ± SD) and 102 men (24.8%) aged 31.69 ± 9.62 (mean ± SD). The most often indicated place of residence was urban location (≥ 150,000 inhabitants) – 233 participants (56.6%). Almost half of the total number of participants (*N* = 204, 49.5%) of the study were those holding a higher education degree (university or college). Demographic details of the studied population of Polish CCM singers are presented in Table 1.

**Table 1:** Demographic characteristics of the studied CCM singers population.

Gender		Age
Female (*N* = 310)	Mean	28.28
	SD	8.18
	Min	18
	Max	56
Male (*N* = 102)	Mean	31.69
	SD	9.63
	Min	18
	Max	70
Total (N = 412)	Mean	29.13
	SD	8.68
	Minimum	18
	Maximum	70
Location		**Number of participants (%)**
Urban (≥ 150,000 inhabitants)		233 (56.6%)
Suburban (50000–150,000 inhabitants)		116 (28.2%)
Rural (≤ 50,000 inhabitants)		63 (15.3%)
General education level		**Number of participants (%)**
Primary		16 (3.9%)
Lower Secondary and Vocational		20 (4.9%)
Upper Secondary		172 (41.7%)
Higher (University/College Degree)		204 (49.5%)

Most of the surveyed population were non-smokers—290 participants (70.4%), 71 (17.2%) were current smokers, and 53 (12.9%) were former smokers. Within the smokers group the history of smoking was 8.85 ± 7.02 (mean ± SD) years. On average smoking history was longer in men—11.29 ± 7.93 (mean ± SD) years than in women—7.00 ± 5.66 (mean ± SD) years. These differences were statistically significant (*p* = 0.006). The mean number of cigarettes smoked daily was 9.56 ± 6.12 for the smokers’ group. Men reported a greater number of cigarettes smoked daily 12.61 ± 7.071 (mean ± SD) in comparison with women—7.19 ± 3 0.97 (mean ± SD). These results were statistically significant (*p* < 0.001).

In terms of alcohol consumption 85 (20.6%) respondents admitted they never drink alcohol. The majority of the group (*N* = 302, 72.8%) reported alcohol consumption occasionally (no more than once per week or a few times each month) and 27 respondents (6.6%) admitted to regular consumption (3–4 times a week or more). The proportion of regular alcohol consumption was significantly higher for men than women (*p* < 0.05).

Over 70% (*N* = 316) of respondents expressed interest in the proposed voice test at a later stage of the study and left their contact details to arrange an appointment. Since it was clearly stated that it is addressed to CCM singers specifically, the interest in participation may be indicative of the fact that the singers who answered the survey met the inclusion criteria.

### 3.2 Singing involvement characteristics and achievements

Based on the declared lifetime singing involvement (in years) almost half of the participants (*N* = 187, 45.4%) declared their singing experience was over 10 years. The majority of the respondents reported daily singing time of 1 or 2 h, 52.7 and 33.3% singers, respectively. Only a few cases of individuals who devoted 5 or more hours per day to singing were recorded (*N* = 7, 1.7%). Solo singing was represented by the majority of respondents (*N* = 262, 63.6%), although 131 singers (31.8%) also participated in group singing such as a choir or a folklore group.

When asked about vocal training 283 (68.7%) participants admitted they had received some form of vocal training throughout their singing career.

The majority of the surveyed singers use a microphone during performances (*N* = 305, 74%). Detailed information on singing involvement characteristics is presented in Table 2.

**Table 2:** Numeric and percentage distribution of the singers regarding singing involvement characteristics.

		Number of participants (%)
Lifetime singing involvement (in years)	<1 year 1–2 years 2–5 years 5–10 years >10 years	12 (2.9) 33 (8.0) 63 (15.3) 117 (28.4) 187 (45.4)
Daily singing time (in hours)	1 h 2 h 3 h 4 h 5 h >5 h	217 (52.7) 137 (33.3) 34 (8.3) 10 (2.4) 7 (1.7) 7 (1.7)
Solo/ Group singing	Solo Group Solo + Group	262 (63.6) 19 (4.6) 131 (31.8)
Vocal training	None Some form of / random vocal training (a single lecture or a group class) A few individual classes Regular individual classes	129 (31.3%) 35 (8.3%) 42 (10.2%) 206 (50%)
Use of microphone	Yes Sometimes No	305 (74.0) 77(18.7) 30 (7.3)

The singers were encouraged to provide multiple responses regarding the musical genres they performed, if appropriate. Three hundred (73%) of the surveyed CCM singers performed in a particular genre, with pop being the dominant one (*N* = 218). Being a solely pop singer was declared by 81% of women and 43% of men. Pop and rock were the most frequently mentioned genres, with 296 (71.8%) and 89 (21.6%) respondents, respectively. A detailed breakdown of the indicated styles is presented in Table 3.

**Table 3:** Numeric and percentage distribution of the singers regarding indicated styles of music performed.

Indicated styles of music performed*	Number of responses (%)
Pop	296 (71.8)
Rock (incl. Classic, alternative, indie, progressive)	89 (21.6)
Gospel/Religious (incl. Traditional, contemporary, praise and worship)	41 (10.0)
Jazz	37 (9.0)
Metal	22 (5.3)
Soul/ R’n’B	19 (4.6)
Folk	18 (4.4)
Blues	11 (2.7)
Hip-hop/ RAP	4 (1.0)

*Multiple answers possible.

The surveyed singers usually indicated more than one venue in which they typically perform (Table 4). The most frequently reported ones were small venues, such as bar and pub gigs or promotional events (*N* = 244, 59.2%).

**Table 4:** Numeric and percentage distribution of the singers regarding types of venues in which they typically perform.

Type of venue in which singers typically perform*	Number of responses (%)
Small venues (bar and pub gigs, promotional events)	244 (59.2)
Big concert halls	188 (45.6)
Open-air concerts	204 (49.5)
Studio recordings	198 (48)

*Multiple answers possible.

Asked about participation in a singing contest, over 50% of the respondents expressed the intention of taking part in one. For 47 singers insufficient vocal skills were the reason for not intending to take part in a singing contest.

Taking part in a TV talent show was an intention of only 9.5% of the singers, 46.1% were not interested and 12.1% considered their vocal skills not sufficient for this kind of activity. Detailed response distribution regarding participation in a singing contest and a TV talent show is presented in Table 5.

**Table 5:** Numeric and percentage distribution of the singers regarding participation in a singing contest /TV talent show.

Query: Have you ever taken part in a singing contest / TV talent show?		
Response	Singing contest	TV talent show
Yes, I succeeded	33 (8.0%)	64 (15.5%)
Yes, but I did not succeed	42 (10.2%)	69 (16.7%)
No, I am not interested	82 (19.9%)	190 (46.1%)
No, I do not think my vocal skills are good enough	47 (11.4%)	50 (12.1%)
No, but I am planning to take part	208 (50.5%)	39 (9.5%)

### 3.3 Voice-related occupational profile

**Figure 2:**
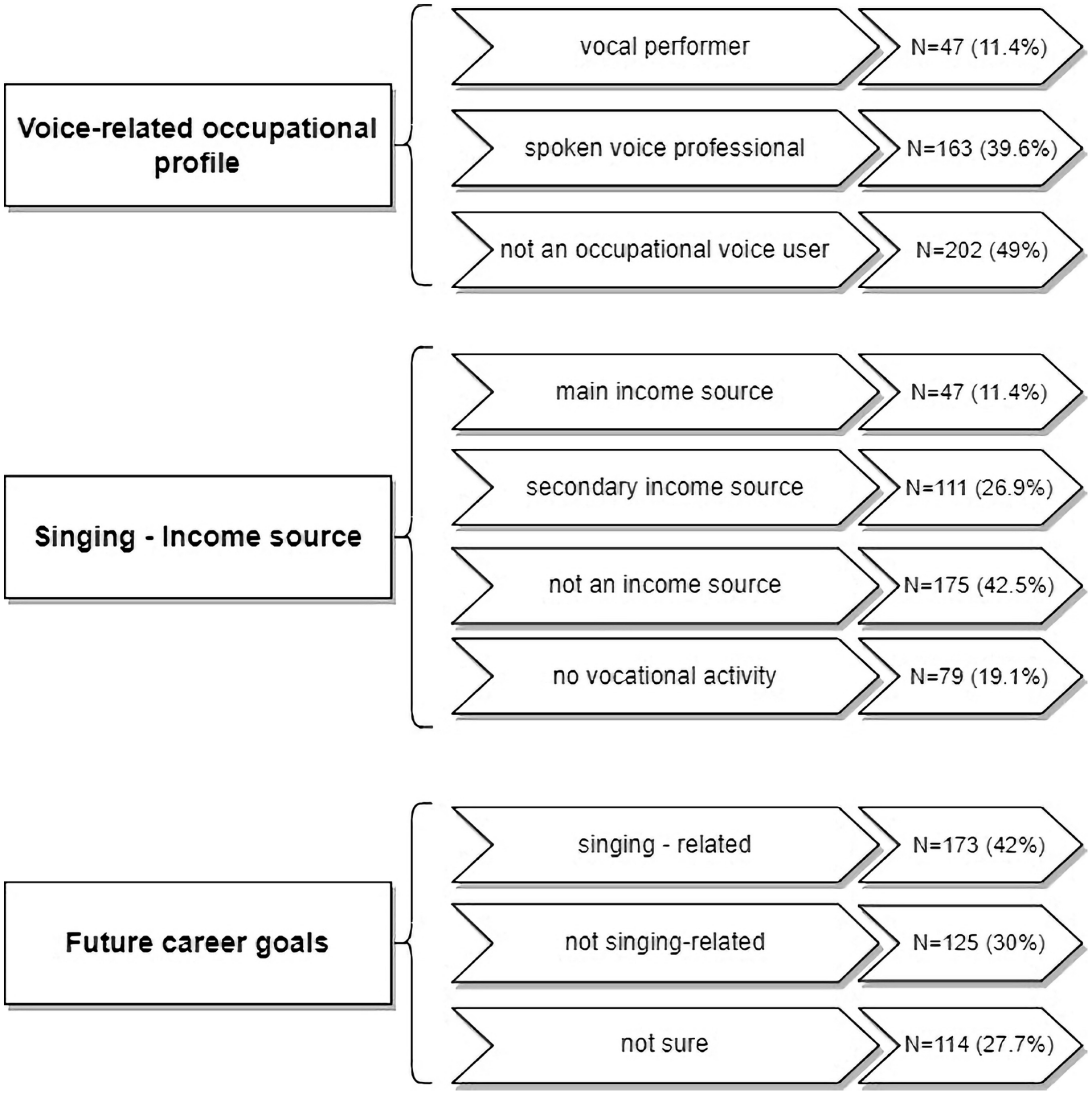
Voice-related occupational profile of the participants of the study.

Half of the participants of the study reported occupational voice use, being either vocal performers (11.4%) or spoken voice professionals (39.6%). In the vocal performer group were singers and actors, while the spoken voice professional group included vocally-demanding jobs like teachers, sales representatives, coaches, trainers, and call center workers. At the time of the survey, 79 (19.1%) participants reported no vocational activity. For 47 singers (11.4%) singing was the primary income source and 173 (42%) defined their future career goals as voice-related. Figure 2 presents a detailed analysis of the voice-related occupational profile of the studied population of CCM singers.

### 3.4 Self-assessment of the speaking voice

#### 3.4.1 Vocal tract discomfort scale

The median total score for the Vocal Tract Discomfort Scale in the studied group of CCM singers was 22.50 (range 0–84). The results were similar for women and men (Table 6). In the Frequency subscale, the median score was 11.00 (0–44). The highest median VTD Frequency value was observed for the group of men at 12.00 (range 1–39). In the Severity subscale, the median score was 12.00 (range 0–40). The highest median VTD Severity value was observed for the group of women at 12.00 (range 0–40). These differences were not statistically significant.

**Table 6:** VTDS scores in the studied CCM singers population.

	N	Mean	Median	SD	SEM	95% Confidence Interval		Min	Max	*p*
						Lower band	Upper band			
VTDS Frequency										
Women	310	13.5	11.00	9.09	0.52	12.48	14.51	0	44	0.659
Men	102	14.03	12.00	9.17	0.91	12.23	15.83	1	39	
Total	412	13.63	11.00	9.10	0.45	12.75	14.51	0	44	
VTDS Severity										
Women	310	12.95	12.00	8.96	0.51	11.95	13.96	0	40	0.803
Men	102	13.28	10.50	9.20	0.91	11.48	15.09	0	36	
Total	412	13.04	12.00	9.01	0.44	12.6	13.91	0	40	
Total VTD										
Women	310	26.45	23.00	17.69	1.0	24.48	28.42	0	84	0.739
Men	102	27.31	22.00	18.19	1.80	23.74	30.89	2	75	
Total	412	26.66	22.50	17.80	0.88	24.94	28.39	0	84	

VTDS, Vocal Tract Discomfort Scale.

#### 3.4.2 Dysphonia symptoms

Nine dysphonia symptoms: hoarseness, dry cough, wet cough, voice breaks, aphonia episodes, shortness of breath while talking, throat clearing, and effortful speaking were analyzed in terms of frequency of occurrence (Table 7). The maximum total score could amount to 36 points. The median total score of dysphonia symptoms for the whole studied population was 12.00 (range 0–27) points. Women reported a lower median score than men with 11.100 (range 0–25) and 12.00 (range 2–27) points for women and men, respectively.

**Table 7:** Dysphonia symptoms scores in the studied CCS population.

		N	Mean	Median	SD	SEM	95% Confidence Interval Lower Upper Band		Min	Max	*p*
		
Hoarseness	Women	310	1.53	1.00*	0.76	0.04	1.44	1.61	0	4	0.035
	Men	102	1.75	2.00*	0.91	0.09	1.58	1.93	0	4	
	Total	412	1.58	2.00	0.80	0.04	1.51	1.66	0	4	
Dry cough	Women	310	1.04	1.00*	0.67	0.04	0.96	1.11	0	4	<0.001
	Men	102	1.42	1.00*	0.94	0.09	1.24	1.61	0	4	
	Total	412	1.13	1.00	0.76	0.04	1.06	1.21	0	4	
Wet cough	Women	310	1.02	1.00*	0.76	0.04	0.93	1.10	0	4	<0.001
	Men	102	1.38	1.00*	0.90	0.09	1.21	1.56	0	4	
	Total	412	1.11	1.00	0.81	0.04	1.03	1.19	0	4	
Aphonia episodes	Women	309	0.71	1.00	0.66	0.04	0.64	0.79	0	3	0.303
	Men	99	0.63	1.00	0.62	0.06	0.50	0.75	0	2	
	Total	408	0.69	1.00	0.65	0.03	0.63	0.75	0	3	
Shortness of breath	Women	310	1.25	1.00	0.94	0.05	1.15	1.36	0	4	0.941
	Men	99	1.23	1.00	0.97	0.10	1.04	1.43	0	4	
	Total	409	1.25	1.00	0.95	0.05	1.15	1.34	0	4	
Throat clearing	Women	310	1.77	2.00*	0.92	0.05	1.66	1.87	0	4	0.049
	Men	102	2.02	2.00*	0.99	0.10	1.82	2.22	0	4	
	Total	412	1.83	2.00	0.95	0.05	1.74	1.92	0	4	
Voice breaks	Women	310	1.55	1.00	0.86	0.05	1.45	1.64	0	4	0.114
	Men	102	1.75	2.00	1.01	0.10	1.55	1.94	0	4	
	Total	412	1.59	2.00	0.90	0.04	1.51	1.68	0	4	
Vocal fatigue after voice use	Women	310	1.58	1.00*	0.96	0.05	1.48	1.69	0	4	0.003
	Men	102	1.89	2.00*	0.96	0.09	1.70	2.08	0	4	
	Total	412	1.66	2.00	0.97	0.05	1.57	1.75	0	4	
Effortful speaking	Women	310	0.65	1.00	0.72	0.04	0.57	0.73	0	4	0.672
	Men	102	0.64	1.00	0.77	0.08	0.49	0.79	0	4	
	Total	412	0.65	1.00	0.73	0.04	0.58	0.70	0	4	
Dysphonia Symptoms Total Score	Women	310	11.10	11.00*	4.63	0.26	10.58	11.61	0	25	0.022
	Men	102	12.66	12.00*	5.58	0.55	11.56	13.75	2	27	
	Total	412	11.48	11.00	4.92	0.24	11.01	11.96	0	27	

*Statistical significance.

**Figure 3:**
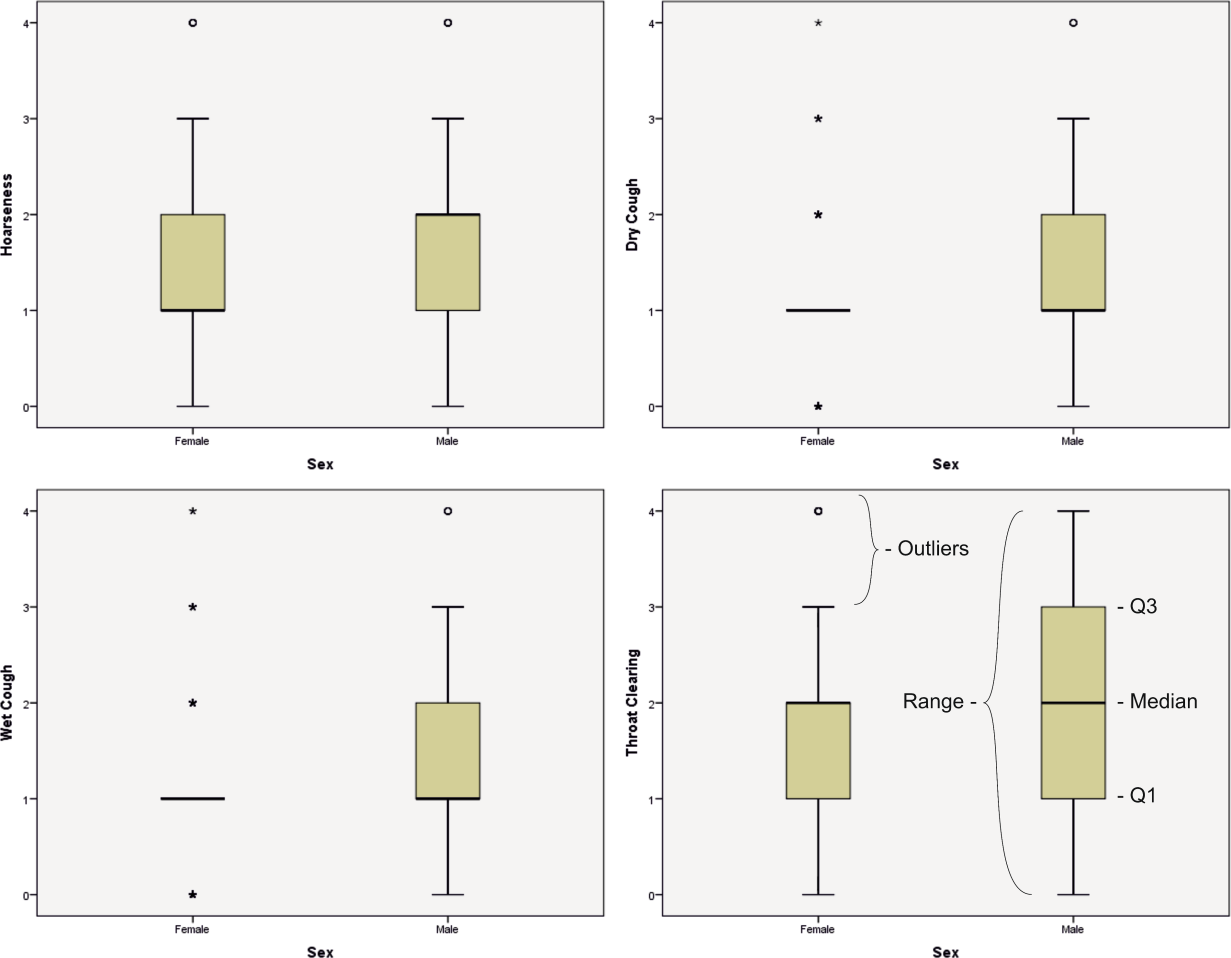
Box-whisker plots for the highest-rated dysphonia symptoms.

Statistically significant differences between men and women were observed for the following variables: hoarseness (*p* = 0.035), dry cough (*p* < 0.001), wet cough (*p* < 0.001), and throat clearing (*p* = 0.049), vocal fatigue after voice use (*p* = 0.003), and for the Total Symptoms Score (*p* = 0.022; Table 7). The highest-rated symptoms, that is the most frequently occurring were throat clearing, vocal fatigue after voice use, voice breaks and hoarseness, all with median values of 2.00 (range 0–4). Figure 3 presents box-whisker plots for the highest-rated dysphonia symptoms.

#### 3.4.3 Overall quality of the speaking voice (analogue VAS scale)

In the assessment of the speaking voice quality using an analogue VAS scale, the median score for the Total study group was 8.00 (range 0–10; Table 7).

### 3.5 Self-assessment of the singing voice

#### 3.5.1 Singing voice handicap index

Median scores of the Singing Voice Handicap Index were determined for the total Study Group and for women and men separately (Table 8). The median score of the SVHI for the total Study Group was 33 (range 0–139). This value exceeds the normative value for the SVHI of 20.35 reported in the literature (54). The scores ranged from 0 to 139. The median SVHI scores were higher for women than men with 34.00 ± 24.77(range 0–139) and 30.00 (range 3–139) points, respectively. All the reported SVHI results exceeded the suggested threshold of the norm (Table 9).

**Table 8:** VAS speaking scores in the studied CCS population.

VAS Speaking	N	Mean	Median	SD	SEM	95% Confidence Interval		Min	Max	*p*
						**Lower band**	**Upper band**			
Women	310	7.77	8.00	1.70	0.09	7.58	7.96	0	10	0.068
Men	102	7.48	8.00	1.51	0.15	7.18	7.78	3	10	
Total	412	7.70	8.00	1.66	0.08	7.54	7.86	0	10	

VAS, Visual Analogue Scale.

**Table 9:** SVHI scores in the studied population of CCM singers.

SVHI TOTAL	N	Mean	Median	SD	SEM	95% Confidence Interval		Min	Max	*p*
						Lower band	Upper band			
Women	310	38.74	34.00	24.77	1.41	35.97	45.51	0	139	0.792
Men	102	38.0	30.00	23.47	2.32	33.41	42.63	3	139	
Total	412	38.56	33.00	24.43	1.20	36.19	40.93	0	139	

SVHI, Singing Voice Handicap Index.

Looking at scores of the individual items of the SVHI, the four highest scores were observed for statements #1 – *It takes a lot of effort to sing*, #5 - *My ability to sing* var*ies day to day,* #15- *I have no confidence in my singing voice* #29- *My singing voice tires easily.* For all these statements the median score was 2.00 (range 0–4).

#### 3.5.2 Overall quality of the singing voice (analogue VAS scale)

In the assessment of overall singing voice quality using the analogue VAS scale, the median scores were 7.00 (range 1–10) and 6.50 (range 3–9) for women and men, respectively. These values were lower than the values in the similarly tested speaking voice quality, indicating that the respondents were less satisfied with the overall voice quality in singing.

### 3.6 Correlations

The correlation analysis for the studied variables showed a weak, statistically significant negative correlation between lifetime singing involvement and the SVHI scores (*r* = −0.132, *p* = 0.007), meaning that the more years the singers have been singing, the lower the scores of the SVHI.

Weak, statistically significant positive correlations were observed between the duration of the singing career and VAS Speaking (*r* = 0.141, *p* = 0.004) and VAS Singing (*r* = 0.177, *p* < 0.01), indicating that longer experience in singing increases the singers’ overall satisfaction with voice, both in speech and singing.

There was no correlation for years of smoking and Dysphonia Symptom scores.

Looking at the strength of the association between alcohol consumption and the results of the self-assessment scales used in the study, positive correlations were found between this variable and VTDS (*r* = 0.128, *p* < 0.01), Voice Symptoms (*r* = 0.1; *p* = 0.042). A weak negative correlation was found between alcohol consumption and the self-perceived overall voice quality in singing reported in the VAS scale.

There were statistically significant, but weak correlations between vocal training and total scores of all the results of the self-assessment tools in the study. The correlations between vocal training and VTDS, Voice Symptoms, and SVHI are negative, meaning that individuals who participated in some form of vocal training report less voice handicap and fewer voice symptoms. Positive correlations observed between vocal training and VAS Speaking and VAS Singing indicate a greater general satisfaction with voice in singers with vocal training. We did not find any statistically significant correlations between microphone use and the results of any of the self-assessment scales used in the study (Table 10).

**Table 10:** VAS singing scores in the studied CCM singers population.

VAS Singing		N	Mean	Median	SD	SEM	95% Confidence Interval		Min	Max	*p*
							Lower band	Upper band			
Women	310	6.76	7.00	1.78	0.10	6.56	6.96	1	10	0.051	Men	102	6.43	6.50	1.54	0.15	6.13	6.73	3	9	Total	412	6.68	7.00	1.73	0.08	6.51	6.85	1	10	

VAS, Visual Analogue Scale.

There were strong positive statistically significant correlations between both subscales of VTDS and Dysphonia symptoms total score and VTDSa (*r* = 0.767, *p* < 0.001) and VTDSb (*r* = 0.722, *p* < 0.001). These results demonstrate that the throat symptoms reported in VTD are reflected in the reduced quality of vocal function described by commonly used in clinical practice dysphonia indicators.

Assessing the strength of relations between the overall assessment of the speaking voice quality and VTDS, we observed moderate negative statistically significant correlations for both subscales of the VTD: Frequency (*r* = −0.355, *p* < 0.001) and Severity (*r* = −0.360, *p* < 0.001). These results can suggest that more frequent and severe symptoms reported in the VTDS negatively influence the overall assessment of the speaking voice.

In the analysis of correlations between SVHI and self-perceived overall quality of the singing voice (VAS singing), a strong, negative significant correlation was found between these two self-assessment tools (*r* = −0.671, *p* < 0.001). The result indicates that the higher the SVHI scores, the lower the singing voice quality perceived by the singers.

Assessing the strength of relations between the results of self-assessment of the speaking and singing voice, strong positive significant correlations were observed between SVHI and both VTDS subscales: VTDSa (*r* = 0.632, *p* < 0.001) and VTDSb (*r* = 0.611, *p* < 0.001). The correlations show that the greater the frequency and intensity of the vocal tract discomfort symptoms, the greater the self-perceived handicap in singing. We found strong positive significant correlations between VAS speaking and VAS singing scores (*r* = 0.479, *p* < 0.001). It can be assumed that there is a close relation between the perceived satisfaction with the quality of speaking and singing voice. A detailed analysis of the correlations between all the self-assessment tools used in the study is presented in Table 11.

**Table 11:** Correlations between lifetime singing involvement, occupational voice use, smoking, alcohol consumption, vocal training, microphone use, and self-assessment of speaking and singing voice.

		VTDS frequency	VTDS severity	VTDS total	Voice symptoms total	VAS speaking	SVHI	VAS singing
Lifetime singing involvement	rho p	−0.010 0.838	0.013 0.792	0.001 0.983	−0.024 0.621	0.141** 0.004	−0.132** 0.007	0.177** <0.01
Occupational voice use	rho p	−0.070 0.156	−0.065 0.189	−0.068 0.167	−0.014 0.777	0.093 0.060	−0.156** 0.001	0.121* 0.014
Smoking (no/former/present)	rho p	0.072 0.143	0.062 0.210	0.068 0.167	0.109 0.026	−0.003 0.953	−0.023 0.649	−0.044 0.848
Smoking (years)	rho p	0.048 0.328	0.051 0.301	0.050 0.312	0.097* 0.050	−0.015 0.765	−0.028 0.575	−0.039 0.435
Alcohol consumption	rho p	0.120* 0.015	0.132** 0.007	0.128** <0.01	0.100* 0.042	−0.004 0.935	0.058 0.237	−0.109* 0.028
Vocal training	rho p	−0.292** <0.01	−0.258* <0.01	−0.258** <0.01	−0.287** <0.01	0.107* 0.030	−0.249** <0.01	0.284** <0.01
Microphone use	rho p	−0.025 0.615	0.001 0.986	−0.014 0.778	0.015 0.756	0.037 0.450	−0.101* 0.040	0.028 0.569

**Correlation is significant at 0.01 level (2-tailed). *Correlation is significant at 0.05 level (2-tailed). VTDS, Vocal Tract Discomfort Scale; VAS, Visual Analogue Scale; SVHI, Singing Voice Handicap Index.

## 4 Discussion

To date, the subject of CCM singers in Poland has not received enough attention. The only study we were able to identify in the literature addressing voice disorders in CCM singers is by Sielska Badurek et al. (55) in which the authors assessed the voice quality and the vocal tract function in popular singing students (*N* = 45) at the beginning of their singing training at the High School of Music. However, in comparison to the sample surveyed in our study (*N* = 412), this group was much smaller and quite homogeneous in terms of age and population characteristics. In our previous work, we presented a literature review on the subject of CCM singers (55) to raise awareness among healthcare professionals of the special consideration and care CCM singers deserve. In the present research, we sought to study this group on a multifaceted level to get a broad picture of who Polish CCM singers are and to obtain data on their self-perceived voice symptoms and voice quality in speech and singing to determine the prevalence of voice problems in this population. We intended to present a description of this group bearing in mind that a number of factors can influence some vocal aspects related to the singing activity (56). In the present study, we included the following variables: lifetime singing involvement, occupational voice use, smoking, alcohol consumption, vocal training, and microphone use to preliminarily examine whether the presence of vocal symptoms and the general satisfaction with voice is affected by them.

### 4.1 Demographic and singing involvement characteristics

Our study group had a significantly higher percentage of females (75.2%), which is in line with numerous studies (12, 33, 57, 58). There could be several explanations for this fact. For instance, Lu et al. suggested that within the singers’ population women seek health information more often than men (57) while Santos et al. theorize that women are more interested in participating as volunteers in research studies (12). It is reported in the literature as well that there is a predominance of women in the popular singing style (56), however, it should be pointed out that there are many variables, for instance, social and cultural, behind why there are more women than men that work as singers.

The vast majority of the studied group (84.8%) lived in urban and suburban areas. Large and medium-sized cities offer more opportunities to access venues and reach audiences so naturally, such places might facilitate singing activity, especially when it is considered a source of income.

Our research sought information on whether the survey participants underwent vocal training throughout their singing careers. Some popular singers are known to launch their professional careers based solely on the talent they present for singing (12). Since vocal production in popular singing is believed to be closer to speaking, singers consider formal musical training unnecessary and, therefore, start their working life based on their empirical knowledge (56). In our study 283 (68.7%) participants admitted they had received vocal training at some point throughout their singing career, therefore contradicting this popular belief. Since a detailed analysis of the nature of the singing instruction received was beyond the scope of this study, these numbers should be interpreted with caution. Nevertheless, it should be noted that there exists a vast spectrum of variability with respect to the standard and substance of diverse vocal training programs. That means that the undertaking of singing lessons does not necessarily result in a reduction of the probability of developing dysphonia (59). Individuals who pursue private singing instruction frequently encounter disappointment when conventional classical training methodologies are presented instead of those that feature CCM styles. Due to the emerging nature of CCM pedagogy and the limited recognition of distinguished pedagogues in this domain, the independent vocalist seems to be challenged to have the responsibility of identifying a reliable source of learning (60).

According to our assumptions, most of the surveyed singers (*N* = 305, 74%) reported using the microphone during performances. The use of electronic amplification of sound is one of the differences between the performances of CCM singers and classical singers. The operatic technique centers on the cultivation of an exceptional vocal range, one that can reach and sustain a wide range of pitch and dynamics. It is further aimed at the projection of the singer’s voice in large concert halls and theaters, where the accompaniment by orchestral music is almost always present, requiring the vocalist to project their voice with utmost clarity and strength without amplification (12). This difference should also be taken into account when offering vocal training to CCM singers. As Titze noticed, the training regimen for the amplified singer revolves around developing a vast collection of predictable and manageable vocal sounds at low levels of acoustic power whereas the training program for the unamplified singer centers on perfecting a limited set of sound combinations to achieve an optimal output of acoustic power (61).

The investigation conducted by our study has brought forward the matter of additional pursuits that are undertaken by artists, including their involvement in singing competitions or their appearances on television broadcasts showcasing their talents. The literature lacks empirical evidence that illustrates the aforementioned issues regarding CCM singers. Consequently, it is infeasible to perform comparative analyses of across-population data. Nonetheless, there exist noteworthy findings that deserve attention. First, we delineated between singing competitions and TV talent shows. In Poland, the former commonly occurs on a localized scale, with limited publicity and often capturing the attention of only the most devoted individuals, i.e., singers. On the other hand, it is the shows like IDOL, X-Factor, Got Talent, The Voice, and The Masked Singer (62) that have gained popularity all over the world over the past two decades. They lure the prospective participants with the promise of subsequent fame to winners, opportunities of securing recording contracts, and a chance at celebrity (63). Engagement in such programs can be perceived as an expedited route toward pursuing a career in singing. Moreover, the medium of television has the potential to facilitate the advancement of singers who possess well-established assets, such as albums or singles that are available through online vendors or physical distribution channels, by enhancing their visibility and augmenting their sales figures toward a wider consumer base (63). At this point, it is noteworthy to emphasize that 19.4% of the survey respondents acknowledged that their artistic output has been formally recorded by a recording company and is presently commercially available. Based on the survey results, it can be inferred that singing competitions hold a greater degree of significance and regard among the participating singers. This is evidenced by the fact that half of the surveyed individuals (50.5%) expressed their intention to partake in such competitions. When making a comparison, it was revealed that merely 9.5% of participants indicated a willingness to engage with the television program. When queried regarding their involvement in competitions and programs to date, successful or not, 32% of participants indicated their participation in a television show, while a smaller number reported participation in the competition (18.2%). In both cases, singing competitions and TV talent shows, the participants of the study exhibit an understanding of the necessity of adequate vocal proficiency for attaining success as 11.4 and 12.1% of the respondents felt that their skills were not sufficient to participate in a singing competition and TV talent show, respectively. The aforementioned data potentially offer a meaningful contribution to the ongoing discourse regarding vocal pedagogy, with a particular focus on CCM singers, by aiding in the identification and communication of pertinent needs for this population.

Another issue we intended to explore in the survey was the occupational profile of the participants. Based on the literature findings, we took into consideration the fact that daily vocal load and overall usage contribute to the occurrence and persistence of vocal disorders (64). Half of the surveyed cohort of CCM singers were occupational voice users. Vocal demands in voice-based professions vary and so does daily vocal load. In some professions, for instance in teachers, there is a high vocal load, but the voice quality does not need to be superb. In other professions, such as journalist the vocal load might not be very high, but vocal excellence is of utmost importance. In the case of singers, the demands on the voice and vocal load are high. Wilson suggests, therefore, that for the performers an ideal kind of “day job” should be one that is possibly most undemanding (65).

### 4.2 Self-assessment of voice

The prevalence of voice problems in the surveyed cohort is based on the participants’ self-assessment. Even though the role of objective measurements in diagnosing voice disorders is indisputable, they are inefficient in quantifying patients’ subjective perceptions of the extent of the disorder and its impact on the overall quality of life (52). For this reason, self-perception of voice disorders has gained importance, and over the past years, a number of significant research tools have been developed based on it (10). Whereas singers may report general complaints about their voice, they are also likely to report complaints that are specific to their singing voice (41). Therefore, we asked the participants to evaluate the quality of their voices both in speech and in singing.

For the self-assessment of the speaking voice, we used the Polish version of the Vocal Tract Discomfort scale. Given the absence of universally agreed-upon normative values for the VTD scale, our investigation drew comparisons between the outcomes we obtained and those reported in prior studies available in the literature (66–68). In a study conducted on a general population, Robotti et al. (66) reported mean VTDS scores of 15.3 ± 8.7 for the Frequency subscale and 15.5 for the Severity subscale in a group of dysphonic individuals. Darawsheh et al. (67) applied the VTDS scale in the diagnosis of voice disorders in singers. The authors examined 97 participants (31 student singers and 66 non-professional voice user students) and reported scores of 14.3 ± 9.9 for the Frequency subscale and 13.3 ± 9.8 for the Severity subscale for the student singers. Our results, being in line with these data might corroborate the fact that singers, being professional voice users, demonstrate increased vocal load to achieve their high-demand task of singing (67, 68). Additionally, we related the total VTDS scores reported by CCM singers in our study to the results presented by Lukaschyk et al. who set normative values for the German VTDS. They proposed VTDS ranges as follows: no (score: 0–13), mild (score: 14–26), moderate (score: 27–40), and severe (score: 41–96) disorder (68). In light of these data, the CCM singers with the VTD mean total score of 26.66 ± 17.60 fall into the mild/moderate voice disorder category. In a study in 2012, Niebudek Bogusz et al. (49) reported the VTDS Frequency and Severity subscales mean values of 23.5 points and 24.6 points, respectively, in a group of professional voice users with voice disorders. These results were much higher than the results in our research. However, unlike in the cited study, only half of the participants in our study were occupational voice users, and voice disorders were not confirmed objectively in CCM singers.

In the process of research design, we concluded that the VTDS scale itself does not fully encompass the topic of voice symptoms in speech. Therefore, we examined the frequency of occurrence of dysphonia symptoms including nine typical voice complaints not included in VTDS. The presence of throat symptoms and dysphonia symptoms reported by the participants of our study confirms what has been reported in the literature, that is that singers can present diverse complaints concerning their voice, such as hoarseness, loss of voice, coughing, pain in the neck region, as well as a feeling of a lump in the and dryness. These sensations can be caused by a lack of understanding of vocal anatomy and physiology, the inappropriate use of the voice, and a lack of knowledge of specific vocal techniques and training for use with the singing voice and they can compromise the voice-related quality of life of singers.

Probably the most important finding of our research is the result of self-assessment of voice in singing reported in SVHI. The mean score for the total study group was 36.19 ± 24.43 (mean ± SD) which is higher than those reported in other studies. Relating these results to the SVHI cut-off value of 20.35 points SVHI determined by Sobol et al. in a meta-analysis (54), the number of participants whose SVHI results exceeded the threshold of the norm amounts to 73.3%. In a study examining 47 healthy professional singers, Castelblanco et al. reported a mean SVHI score of 22.4 points. The subjects in this study specialized in the genres of classical solo, choral, and opera solo (17). Baracca et al. conducted their research on 214 singers with singing styles categorized as classical or modern. The SVHI scores in healthy singers were significantly lower (29.26 ± 25.72) than those obtained in the group of singers with a vocal fold abnormality (45.62 ± 27.95). However, the authors did not report whether there were differences in the scores obtained by classical and modern singers (69). Lloyd et al. in their research in 2019 compared the prevalence of vocal fold pathologies among first-year singing students from the classical (*N* = 19), musical theater (*N* = 15), and CCM (*N* = 23) genres. They used a shortened version of the Singing Voice Handicap Index - SVHI -10 and reported differences between CCM and classical singers, with CCM singers demonstrating greater singing voice handicap. Another study focused on the assessment of singing voice handicap in students is that by Sielska Baduek et al. (70) who examined the vocal health of 45 CCM singing students. The mean scores obtained by the study participants (21.8 ± 15.2) remained within the normal range. Interestingly, it was also reported that 22 % of CCM singing students began their education with vocal nodules, despite showing a self-perception of voice disorders within a normal range. Given the heterogeneity of the sample of singers in our study and the high scores observed in SVHI, it might be inferred that in singers, amateurs, or professionals, any change in the singing voice can contribute to a deterioration in their quality of life. This, in turn, may lead to consequences on both the functional and emotional side, as reported in SVHI.

Looking at the discrepancy between the SVHI results in the studies conducted by other authors and ours, an important question arises: why are the results obtained in the SVHI by CCM singers from our cohort so high, and should they be interpreted as alarming? One of the reasons for such high scores could be the fact that popular singers have their own style, often based on imitation, which makes them more susceptible to vocal abuses (58).

Although there is evidence in the voice literature that women are more likely than men to experience vocal health problems (71) our study did not confirm that. The number of women participating in the survey does not translate into worse results in self-assessment of the singing voice. A similar finding was reported by Renk et al. who compared the differences in scores between the VHI-10 and the SVHI-10 in a group of 50 singers (41). It could be assumed that men with voice problems were more likely to answer the questionnaire. On the other hand, male singers may be more aware of their voice and sensitive to voice changes than male non-singers (72). Mathman et al., in turn, concluded that the fact that older singers reported fewer complaints than younger singers could be also attributed to older singers acquiring strategies to avoid health-damaging behavior (73). Other factors that could contribute to SVHI scores exceeding the threshold of the norm could be poor technical preparation, incorrect repertoire selection, and, as pointed out by Rosa and Behlau, wrong vocal classification, incorrect use of voice, and lack of vocal training on warm-up and cool-down techniques (53). A detailed analysis of the factors that were not included in our study would probably shed more light on the subject and provide more insight into the aspect of occupational health and safety in professional CCM singers.

Assessing the correlations between the scores of self-assessment scales and the studied variables, we found statistically significant correlations for the SVHI and 3 variables: lifetime singing involvement (*r* = −0.132, *p* = 0.007), occupational voice use (*r* = −0.156, *p* = 0.001), vocal training (*r* = −0.249, *p* < 0.01). The correlation between SVHI and vocal training, as well as high SVHI scores indicating a great degree of singing voice handicap, are in agreement with the conclusion drawn by Sielska-Badurek et al. that proper CCM singing technique acquisition is extremely important as it may prevent vocal trauma (70). It was beyond the scope of this research to examine in detail the duration and quality of vocal training received by the survey participants, however, this notion is worth exploring in future research. The correlation between lifetime singing involvement and SVHI scores may be indicative of the fact that more years of singing experience might help in terms of the lower impact the voice problems and symptoms generate.

Since self-assessment of voice in our study concerned both speech and singing, we looked at the relationship between the two. We found statistically significant correlations between both subscales of VTD and SVHI. This result is in line with the study by Pinheiro et al. who examined 100 church gospel singers and found significant correlations between the VTD scale and Modern Singing Handicap Index (MSHI) (74) (Table 12).

**Table 12:** Correlations between the results of self-assessment tools: VTD, Dysphonia symptoms, VAS Speaking, SVHI, VAS singing used in the study.

		VTDSa	VTDSb	Dysphonia symptoms	VAS Speaking	SVHI	VAS Singing
VTDSa	rho p	1.00 <0.001	0.927^*^ <0.001	0.767^*^ <0.001	−0.355^*^ <0.001	0.632^*^ <0.001	−0.521^*^ <0.001
VTDSb	rho p	0.927^*^ <0.001	1.00 <0.001	0.722^*^ <0.001	−0.360^*^ <0.001	0.611^*^ <0.001	−0.489^*^ <0.001
Dysphonia symptoms	rho p	0.767^*^ <0.001	0.722^*^ <0.001	1.00 <0.001	−0.314^*^ <0.001	0.527^*^ <0.001	−0.443^*^ <0.001
VAS speaking	rho p	−0.355^*^ <0.001	−0.360^*^ <0.001	−0.314^*^ <0.001	1.00 <0.001	−0.460^*^ <0.001	0.479^*^ <0.001
SVHI	rho p	0.632^*^ <0.001	0.611^*^ <0.001	0.527^*^ <0.001	−0.460^*^ <0.001	1.00 <0.001	−0.671^*^ <0.001
VAS Singing	rho p	−0.521^*^ <0.001	−0.489^*^ <0.001	−0.443^*^ <0.001	0.479^*^ <0.001	−0.671^*^ <0.001	<0.001 <0.001

*Correlation is significant at 0.01 level (2-tailed). VTDSa, Vocal Tract Discomfort Frequency subscale; VTSDb, Vocal Tract Discomfort Severity subscale; SVHI, Singing Voice Handicap Index; VAS, Visual Analogue Scale.

To the best of our knowledge, this is the first study addressing the topic of CCM singers in Poland in such a broad scope. We believe that the data obtained in this initial exploratory study will have practical application in that it will facilitate the evaluation and management of patients who sing CCM vocationally or avocationally. The results provided by this research offer potentially useful information and a framework for subsequent analysis.

### 4.3 Limitations and future directions

The study design did not include instrumental ENT and phoniatric examination, so the presented results and conclusions are based on the subjective self-report voice assessment.

A possible sampling bias in terms of sample representativeness and generalization should be considered since it is not possible to know, given the results, if the singers who agreed to complete the survey presented some difficulty that led them to be interested in participating in the study. Since this study was conducted online, it was limited to participants who had internet access.

There are also several aspects revealed by this study that, if analyzed in detail could provide a more precise explanation of the issue of voice problems reported by singers, but were not taken into account in the design of the study. These are for instance the nature of vocal training received (the duration of the training, the institution where it was held, in what specific CCM style), daily occupational vocal load, other vocal hygiene and health-related aspects such as proper hydration, amount of sleep, allergies, reflux, respiratory diseases. Additionally getting more detailed information on factors such as the type of microphone used, and the venues in which the singers perform would allow for a more accurate profile of this population in occupational health framework. While the group sizes regarding the type of music performed did not provide sufficient power for sub-group analysis, this would be an area for future inquiry as well.

## 5 Conclusion

The participants involved in the study exhibited diversity with regard to their musical genre preferences, aspirations pertaining to singing endeavors, career affiliations, and source of income. A considerable degree of singing voice handicap reported in the SVHI demonstrates that Polish CCM singers are a risk group for developing voice disorders. Voice symptoms reported in Vocal Tract Discomfort Scale and Dysphonia symptoms indicate the need to examine CCM singers’ voice not only in the context of singing but also at the level of the speaking voice. A more in-depth analysis of the variables potentially influencing the degree of self-reported voice problems is necessary, to present an accurate profile of CCM singers in the Occupational Health and Safety framework.

## Data availability statement

The raw data supporting the conclusions of this article will be made available by the authors, without undue reservation.

## Author contributions

JM: Conceptualization, Data curation, Investigation, Visualization, Writing – original draft. WP: Writing – review & editing. PP: Formal analysis, Writing – review & editing. EN-B: Supervision, Writing – review & editing.

## Footnotes

^1^https://www.statisticshowto.com/probability-and-statistics/find-sample-size/
